# Abdominal Regional Fat Distribution on MRI Correlates with Cholecystolithiasis

**DOI:** 10.1371/journal.pone.0109776

**Published:** 2014-10-13

**Authors:** Yang Zhang, Tian Wu Chen, Xiao Ming Zhang, Yi-Xiang Wang, Xiao Xiao Chi, Xing Hui Li, Xiao Feng Gao, Yi Fan Ji

**Affiliations:** 1 Sichuan Key Laboratory of Medical Imaging, Department of Radiology, Affiliated Hospital of North Sichuan Medical College, Sichuan Province, China; 2 Department of Imaging and Interventional Radiology, Faculty of Medicine, The Chinese University of Hong Kong, Prince of Wales Hospital, New Territories, Hong Kong SAR, China; 3 Department of Preventive Medicine, North Sichuan Medical College, Nanchong, Sichuan Province, China; University of Leicester, United Kingdom

## Abstract

**Aims:**

To determine whether abdominal regional fat distribution pattern on MRI is correlated with cholecystolithiasis.

**Methods:**

Magnetic resonance imaging (MRI) of 163 patients with cholecystolithiasis and 163 non-cholecystolithiasis control subjects admitted to our institution between March 2011 and September 2013 were included in this cross-sectional evaluation. There were 98 women and 65 men in cholecystolithiasis group with an average age of 57±16 years (range 25–86 years). There were 87 women and 76 men in the control group with an average age of 41±16 years (range 14–77 years). Visceral adipose tissue (VAT), abdominal subcutaneous adipose tissue (SAT) and total abdominal adipose tissue (TAT) of all the subjects at navel level were measured on abdominal MRI. According to the visceral adipose area (cut-off point VAT = 100 cm^2^), study subjects were divided into 1) increased accumulation of intra-abdominal fat and 2) normal distribution of intra-abdominal fat. Logistic regression was used to assess the association of fat with the presence of cholecystolithiasis, adjusted for age and sex.

**Results:**

The incidence of increased intra-abdominal fat accumulation in the cholecystolithiasis group was significantly higher than that of the control group (P = 0.000). After adjusting for age and sex, cholecystolithiasis was associated with a one standard deviation increment in the waist circumference (WC) (OR = 1.44; 95%CI: 1.01,1.93; p = 0.00), VAT (OR = 4.26; 95%CI: 1.85,5.29; p = 0.00), VAT/SAT (OR = 8.66; 95%CI: 1.60,12.63; p = 0.00), and VAT/TAT (OR = 6.73; 95%CI: 4.24,12.18; p = 0.00), but not with fat content in the abdominal subcutaneous fat (p = 0.19).

**Conclusions:**

The visceral adipose tissue and distribution proportion of abdominal adipose tissue are correlates of cholecystolithiasis.

## Introduction

Gallstone represents a major disease burden, affecting approximately 10–20% of the U.S. population [1]. In China and some other Asian countries, gallstone disease is also an important public health problem [2], [3]. Cholecystolithiasis is very common and can be divided into symptomatic and asymptomatic clinically. Common complications of gallbladder stone include acute cholecystitis, biliary obstruction, acute pancreatitis and cholangitis. Severe complications of cholecystolithiasis include gallbladder perforation, Mirizzi syndrome and fistula formation, and are usually associated with significant morbidity and mortality [4]–[7]. In addition, cholecystolithiasis is strongly associated with gallbladder cancer and considered an intermediate step in gallbladder cancer pathogenesis [8]. In China, there has been an increase in cholesterol stone and a decrease in pigment stone during the past a few decades [9], probably related to increasing obesity and a more westernized diet (containing more fat) and lifestyle (physically inactive) [10]. A large number of studies also show that overweight and obesity are well-established risk factors for gallstone diseases such as cholecystolithiasis [2], [11]–[14]. Central obesity, measured by waist-to-hip ratio, is independently related to risk for gallstone after taking into account total adiposity, as measured by body mass index (BMI) [15]–[16]. High central obesity and BMI are two independent risk factors for metabolic conditions, such as insulin resistance, reduced number of insulin receptors, and low plasma high-density lipoprotein cholesterol and therefore may play an important role in the etiology of gallstone disease [17]. Similarly, there is a significant association between abdominal adiposity and the incidence of symptomatic gallstone disease [14]. However, whether abdominal regional fat distribution is associated with cholecystolithiasis has not been recorded in the reported studies.

Magnetic resonance imaging (MRI) can accurately quantify the volumes of visceral adipose tissue (VAT) and subcutaneous adipose tissue (SAT) [18]–[20]. This study investigated whether distribution characteristics and the quantity of abdominal adipose tissue measured by MRI are associated with cholecystolithiasis.

## Subjects and Methods

### 1 Study population

This study was approved by our institutional review board and the Ethics Committee of Affiliated Hospital of North Sichuan Medical College. Because of the retrospective nature of this study and the informed consent was waived. The study complies with the ethical principles of the Helsinki Declaration of 1964, revised by the World Medical Organization in Edinburgh in 2000. Inpatients with cholecystolithiasis from March 2011 to September 2013 in our hospital who had abdominal MRI were candidates of this study. Non-cholecystolithiasis patients who had abdominal MRI during the same period were used as control subjects.

The inclusion criteria for cholecystolithiasis group were as follows: (1) cholecystolithiasis was confirmed by surgery and pathology; (2) Abdominal MRI was performed after admission and 1 week before surgery and image quality met diagnostic requirements.

For the control group, the inclusion criteria were as follows: (1) US or CT showed no cholecystolithiasis in the patients (2) Abdominal of MRI was performed after admission and showed no cholecystolithiasis. (3) Adipose tissue was displayed clearly on MRI.

The exclusion criteria for both cholecystolithiasis group and non-cholecystolithiasis control group included conditions which can cause the redistribution of the body fat, such as history of chronic consumptions disease, history of using cortical hormone. Finally, 163 patients with cholecystolithiasis and 163 non-cholecystolithiasis control subjects were included in this study. There were 98 women and 65 men in cholecystolithiasis group with an average age of 57±16 years (range 25–86 years). There were 87 women and 76 men in the control group with an average age of 41±16 years (range 14–76 years).

### 2 MRI techniques

All MR images were obtained with a 1.5T whole body scanner (Signa, GE Medical Systems, Milwaukee, WI, USA). The general MRI sequences for abdomen included: transverse gradient echo T1-weighted image (GRE T1WI); cross-sectional respiratory triggered axial fast recovery fast spin-echo T2-weighted image (FRFSE T2WI); coronal and cross-sectional single shot fast spin echo T2-weighted image (SSFSE T2WI); double echo chemical displacement fast spoiled gradient echo (FSPGR), in-phase and out-phase obtained using a breath-hold scanning and every breath-hold was about 20 second; single shot fast spin echo MR cholangiopancreatography (MRCP). T1-weighted imaging was performed using GRE sequence with the following parameters: TR 147 ms, TE 1.7 ms, flip angle 80°, Slice thickness 5∼8 mm, Slice space 0.5∼1.0 mm. T2-weighted imaging was performed using FRFSE with the following parameters: TR 10000∼12000 ms, TE 90∼100 ms, Slice thickness 5 mm, Slice space 0.5 mm. The parameters of double echo with in-phase and out-phase T1 weighted were TR 145 ms, flip angle 75°, matrix 256×192, field of view 42 cm×30 cm, Slice thickness 6 mm, Slice space 1 mm, number of excitation (NEX) 1.

### 3 MR images review

The original MRI data were loaded onto a workstation (Advantage Workstation 4.2; GE Healthcare). The scanning level of navel, L4 or L4/L5 vertebral clearance has been used to study the distribution characteristics of abdominal adipose tissue [21]–[23]. In this study, according to the acquisition of a localizer sequence, the inter-vertebral space between the fourth and fifth lumbar vertebrae was identified by locating the umbilicus [14], [24]–[25].

**Figure 1:**
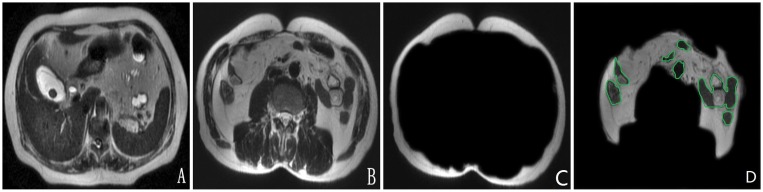
A 52-year-old male with gallbladder stone. One stone can be found in neck of gallbladder (A) and navel level image was found by a localizer sequence (B). SAT is to be the area between outline of abdominal skin and the outer abdominal muscle (C). VAT is to be enterocoelia and retroperitoneal region between the inside edge of abdominal muscles and the spinal front. Intestines and big vessels that have been circled by green line are not included in the intra-abdominal fat area (D). Axial fast-recovery fast spin-echo T2-weighted images without fat suppression shows intra-abdominal fat with SAT = 96.21 cm^2^ and VAT = 104.05 cm^2^.

Based on signals on MR images, the fat area was manually drawn and excluding intestines and big vessels. SAT was defined as the area between outline of abdominal skin and the outer abdominal muscle; VAT was defined as enterocoelia and retroperitoneal region between the inside edge of abdominal muscles and the spinal front. SAT and VAT were divided and measured at navel level on abdominal MRI using area measurement tool (Advantage Workstation 4.2; GE Health-care Technologies, Milwaukee, WI) (Figure 1).

Total abdominal adipose tissue (TAT, TAT = SAT+VAT) and the waist circumference (WC) were also measured and calculated on the images. To examine the reproducibility of the measures of fat depots, the archived scans for the control group were read by 3 different MRI technicians. The technical errors for 3 repeated readings of the same scan by the same observer for SAT and VAT volumes in our laboratory were 0.99% and 1.95% respectively. The intraclass correlation coefficients among the 3 analysts (who each read the same scans twice, separated by a 3-month interval) for SAT and VAT were 0.99 and 0.95 respectively.

### 4 Statistical analysis

This study used Statistical Package for Social Sciences (SPSS) 13.0 software (Chicago, IL, USA) for statistical analysis. All data are presented as mean and standard deviation or n and %. Differences between the two groups were tested for using the t-test. The prevalence of cholecystolithiasis was recorded in subjects with VAT≥100 cm^2^ and VAT<100 cm^2^. A chi-square test was used for the differences in the prevalence of cholecystolithiasis. Spearman correlation coefficients between fat measures were calculated. Logistic regression was used to assess the association of fat measured with the presence of cholecystolithiasis, adjusting for age and sex. The level of significance was set at P<0.05.

## Results

### 1 Demographic and body-composition characteristics

Demographic characteristics of the study subjects and mean values for fat compartments and distribution proportion of the cholecystolithiasis group and the control group are shown in Table 1. Individuals with cholecystolithiasis were older compared to control groups (p = 0.000). Among 163 subjects with cholecystolithiasis, 98(60%) were female. Among 163 subjects in control group, 87(53%) were female. However, this sex differences had no statistical significance. The values of VAT, WC, VAT/SAT, VAT/TAT, TAT of cholecystolithiasis group were substantially higher than those of the control group and the difference had statistical significance, all P = 0.000. However, the difference of SAT between the cholecystolithiasis group and the controls was no significant (P>0.05).

**Table 1:** Subject characteristics and adipose tissue distribution.

Variable	Cholecystolithiasis	Control group	P-value
	(N = 163)	(N = 163)	
Age (years)	57±16	41±16	0.000
Female (n, %)	98(60%)	87(53%)	0.55
WC(mm)	83.48±7.54	77.17±7.35	0.000
SAT(cm^2^)	136.09±94.06	135.06±64.46	0.91
VAT(cm^2^)	112.89±41.06	59.71±21.21	0.000
TAT(cm^2^)	248.96±103.98	194.76±77.06	0.000
VAT/SAT	0.97±0.43	0.49±0.17	0.000
VAT/TAT	0.47±0.10	0.32±0.08	0.000

WC, waist circumference; SAT, subcutaneous adipose tissue; VAT, visceral adipose tissue; TAT, total adipose tissue. Analysis of variance for continuous variables and chi square test for categorical variables.

### 2 The comparison of incidence of intra-abdominal fat accumulation on MRI

Asian with VAT≥100 cm^2^ on CT or MRI can be diagnosed with abdominal obesity [26], [27]. According to this standard, all subjects were divided into 1) increased accumulation of intra-abdominal fat, or 2) normal distribution of intra-abdominal fat. The cholecystolithiasis group and control group for the incidence of intra-abdominal fat increased accumulation were compared. The incidence of intra-abdominal fat increased accumulation in the cholecystolithiasis group was significantly higher than that of control group (p = 0.000, Table 2).

**Table 2:** The incidence of intra-abdominal fat increased accumulation on MRI between the cholecystolithiasis group and the control group.

Groups	VAT≥100 (cm^2^)	VAT<100 (cm^2^)	incidence
Cholecystolithiasis	85	78	52.1%
Control	30	133	18.4%

VAT value for pearson Chi-Square 40.64, P = 0.000.

### 3 Degree of correlation between fat measurements

The VAT, TAT, and WC were all strongly correlated, whereas the TAT was highly correlated with VAT. SAT were moderately correlated with WC. On the other hand, the VAT was weakly correlated with SAT (Table 3).

**Table 3:** Spearman correlation coefficients between abdominal fat measurement on MRI in 326 subjects from the cholecystolithiasis group and the control group.

	WC	SAT	VAT	TAT
WC	1			
SAT	0.61	1		
VAT	0.83	0.34	1	
TAT	0.86	0.81	0.78	1

All p = 0.000.

### 4 The relationship of fat measures with cholecystolithiasis

Logistic regression analysis was used to examine the association of abdominal fat with cholecystolithiasis (Table 4). The VAT, WC, VAT/SAT, VAT/TAT were significantly associated with cholecystolithiasis in single factor logistic regression analysis, whereas the association of the SAT with cholecystolithiasis were rendered statistically non-significant (Model 1). The VAT, WC, VAT/SAT, VAT/TAT were significantly associated with cholecystolithiasis after further adjustment of age (all p = 0.000, Model 2). In age-sex-adjusted models, cholecystolithiasis was associated with a one standard deviation increment in the WC (OR = 1.44; 95%CI: 1.01,1.93), VAT (OR = 4.26; 95%CI: 1.85,5.29), ratio of visceral/subcutaneous adipose tissue (VAT/SAT) (OR = 8.66; 95%CI: 1.60,12.63), and ratio of visceral/total abdominal adipose tissue (VAT/TAT) (OR = 6.73; 95%CI: 4.24,12.18).

**Table 4:** Association of different measurements of abdominal fat and distribution proportion of abdominal adipose tissue with cholecystolithiasis.

Models	OR per SD	P-values
WC*	1.01(1.01,1.03)	0.000
Age-adjusted**	1.01(1.01,1.02)	0.000
Age-sex-adjusted***	1.44(1.01,1.93)	0.000
SAT*	0.77(0.56,1.07)	0.16
Age-adjusted**	0.76(0.54,1.20)	0.17
Age-sex-adjusted***	0.79(0.51,1.11)	0.19
VAT*	5.13(2.39,7.24)	0.000
Age-adjusted**	4.24(1.84,5.16)	0.000
Age-sex-adjusted***	4.26(1.85,5.29)	0.000
VAT/SAT*	9.17(3.62,18.39)	0.000
Age-adjusted**	4.84(1.60,12.63)	0.000
Age-sex-adjusted***	8.66(1.60,12.63)	0.000
VAT/TAT*	6.53(3.97,11.42)	0.000
Age-adjusted**	4.54(2.45,9.37)	0.000
Age-sex-adjusted***	6.73(4.24,12.18)	0.000

Data represent odds ratio and 95% confidence intervals. All abdominal adiposity measurements and ratio are standardized to a mean of 0 and standard deviation of 1.

*Model 1,

**Model 2,

***Model 3.

## Discussion

In our study, the overall burden of abdominal visceral fat and distribution proportion of abdominal adipose tissue on MRI were strongly associated with cholecystolithiasis. We did not find associations of abdominal subcutaneous fat with cholecystolithiasis. To our knowledge, our study is the first to support the presence of associations of the abdominal visceral fat, distribution proportion of abdominal adipose tissue with cholecystolithiasis.

In a previous report, Tsai et al [20] found that there was significant association between abdominal adiposity and the incidence of symptomatic gallstone disease, but they did not discuss the associations of abdominal fat composition and distribution with cholecystolithiasis. Our results showed that waist circumference, visceral adipose tissue, ratio of visceral adipose tissue/abdominal subcutaneous adipose tissue and ratio of visceral adipose tissue/total abdominal adipose tissue were associated with cholecystolithiasis. As measures of abdominal adiposity, waist circumference and waist-to-hip ratio predict the risk of developing gallstones [14]. Similarly, our study demonstrated that larger waist circumference was associated with cholecystolithiasis.

In recent years, authors from Japan and South Korea confirmed that for Asian CT or MRI study of VAT≥100 cm^2^ can be diagnosed with abdominal obesity [26], [27]. The research of Rankinen et al also confirmed that the VAT cut-off point for visceral abdominal obesity was 100 cm^2^ [28]. According to this standard, in our study all subjects are divided into the intra-abdominal fat increased accumulation group and the normal fat distribution group. Our results showed that the incidence of intra-abdominal fat increased accumulation in patients with cholecystolithiasis was significantly higher than that of in control subjects. Our results confirmed that intra-abdominal fat increased accumulation is associated with cholecystolithiasis, which directly supported to the notion that abdominal or centripetal obesity was associated with gallstone disease [2], [11]–[14]. Though the mechanism by which obesity increases the risk of gallstone diseases is still unclear, a few biologically plausible pathways by which central adiposity may be linked to gallstone formation have been proposed [29], [30]. Obesity, particularly abdominal obesity may be correlated with an increased activity of the rate-limiting step in cholesterol synthesis, the hepatic enzyme, 3-hydroxyl-3-methyl-glutaryl co-enzyme A (HMG-CoA) reductase, leading to increased cholesterol synthesis in the liver and its heightened secretion into bile [31]–[33]. High biliary lipid concentration is a factor for the formation of cholecystolithiasis and gallbladder sludge is thought to be the usual precursor of cholecystolithiasis [34].

The occurrence of gallstones disease is positively related to advancing age, as gallstone is unusual in persons younger than 30 years [35]. Age factor has been previously highlighted in several studies. The frequency of gallstones increases with age, escalating markedly after age 40 to become 4 to 10 times more likely in older individuals [2], [36]. The stone type also changes with age: initially being composed predominantly of cholesterol but in late life tending to be black pigment stones [37]. Similarly, autopsy studies conducted in Sweden and the Czech Republic showed the incidence of gallstones to be 30% in men and 50% in women older than 20 years of age [38]. In this study we also further confirmed age was associated with cholecystolithiasis.

In our study, gender was not associated with cholecystolithiasis. This negative finding may be due to the limited sample size. In fact, it has been previously documented in many studies that being female is the single most important non-modifiable cause of gallstones [37], [39]. In a Pakistan study, 85.4% of their gallstones patients were female [40]. The underlying mechanism is female sex hormones. Estrogen replacement therapy is an established risk factor for cholesterol gallstone formation [41].

Our study has several limitations. Firstly, our study was a cross-sectional study; therefore, no causal inferences can be made. Secondly, this study was a single center study with small sample size. In the future multicenter data and larger sample size should be included. Lastly, we did not assess subjects’ blood biochemical composition of the fat; it is possible that composition measures of adiposity may show the association between cholesterol, high-density lipoprotein and cholecystolithiasis.

In conclusion our data suggest that the overall burden of intra-abdominal fat, but not abdominal subcutaneous fat, is associated with cholecystolithiasis. The distribution proportion of abdominal adipose tissue is also a correlate of cholecystolithiasis. These findings expand our knowledge of body composition in a cohort with cholecystolithiasis, which provides a reference for a longitudinal design study of the relationship between visceral fat tissue and cholecystolithiasis.
